# Engineered Decellularized Tendon Matrix Putty Preserves Native Tendon Bioactivity to Promote Cell Proliferation and Enthesis Repair

**DOI:** 10.1155/2023/4665795

**Published:** 2023-11-16

**Authors:** Anna-Laura Nelson, Kelsey M. O'Hara, Philip C. Nolte, Naomasa Fukase, Yoichi Murata, Anna-Katharina Nolte, Johnny Huard, David L. Bernholt, Peter J. Millett, Chelsea S. Bahney

**Affiliations:** ^1^Steadman Philippon Research Institute (SPRI), Center for Regenerative Sports Medicine, Vail, Colorado, USA; ^2^The Steadman Clinic, Vail, Colorado, USA; ^3^Orthopaedic Trauma Institute, University of California, San Francisco (UCSF), San Francisco, CA, USA

## Abstract

Rotator cuff tears are a common soft tissue injury that can significantly decrease function of the shoulder and cause severe pain. Despite progress in surgical technique, rotator cuff repairs (RCRs) do not always heal efficiently. Many failures occur at the bone-tendon interface as a result of poor healing capacity of the tendon and failure to regenerate the native histological anatomy of the enthesis. While allografts are commercially available, clinical use is limited as they do not stimulate tissue regeneration and are associated with a structural failure of up to 40% in re-tear cases. Novel tissue engineering strategies are being developed with promise, but most involve addition of cells and/or growth factors which extends the timeline for clinical translation. Thus, there exists a significant unmet clinical need for easily translatable surgical augmentation approaches that can improve healing in RCR. Here we describe the development of a decellularized tendon matrix (DTM) putty that preserves native tendon bioactivity using a novel processing technique. *In vitro*, DTM promoted proliferation of tenocytes and adipose-derived stem cells with an increase in expression-specific transcription factors seen during enthesis development, *Scleraxis* and *Sox9*. When placed in a rabbit model of a chronic rotator cuff tear, DTM improved histological tissue repair by promoting calcification at the bone-tendon interface more similar to the normal fibrocartilaginous enthesis. Taken together, these data indicate that the engineered DTM putty retains a pro-regenerative bioactivity that presents a promising translational strategy for improving healing at the enthesis.

## 1. Introduction

Tendon and ligament injuries affect approximately 17 million Americans each year and are the second leading cause of musculoskeletal injuries [1]. In 2016, there were over 460,000 rotator cuff surgeries performed in the United States and it was the second most common orthopaedic soft tissue repair procedure performed [2]. Despite significant clinical progress to improve surgical technique and rehabilitation protocols, structural outcomes of rotator cuff repair (RCR) remain statistically poor. Re-tear rates as high as 40% have been noted following standard rotator cuff repairs with failures increasing to 73–94% in massive tears [3–10]. In addition to the higher re-tear rates with increased tear size, there is also an increased incidence of repair failures with increasing patient age [11].

Augmenting repair at the tendon-bone junction may improve RCR as this is where the vast majority of postoperative failures occur [5, 12]. Insertion of the tendon into the bone at the rotator cuff occurs through a fibrocartilaginous enthesis where there is a gradual histological transition from tendon to fibrocartilage to calcified cartilage to bone [13]. Adult entheses do not regenerate following RCR, rather repair typically produces a mechanically weak fibrovascular scar lacking the zone of calcified cartilage [14–16]. A number of critical factors likely contribute to the poor regenerative capacity of the tendon, including a paucity of appropriate tendon progenitor cells, the low vascularity of the fibrocartilaginous enthesis, and limited bioavailability of growth factors that can promote regeneration. Given the poor clinical results of RCR, there is currently a major challenge in the field of orthopaedic medicine to stimulate biological healing and reconstruct the enthesis structure to regenerate a stronger tendon.

Interestingly, while adult tendon tissues heal poorly, neonatal tendons can successfully regenerate following injury in a process termed “scarless healing” [17, 18]. Lineage tracing identified *Scleraxis*- *(Scx*-) positive tendon progenitor cells as the key cellular population enabling neonatal tendon development [19, 20]. Recent work has further observed that co-expression of *Scx* and transcription factor *Sox9* is critical to enthesis development and repair [21, 22]. In adult RCR, these *Scx*^+^/*Sox9*^+^ cells are not recruited to the injury and there is minimal cellular proliferation after injury [18, 21]. In the adult tissue, these *Scx*-lineage tenocytes are not recruited to the injury, and there is very minimal cellular proliferation 3 days after injury [18]. Regulation of tenocyte recruitment and direction of collagen fibrils at the enthesis have been reported to be directly regulated by Transforming Growth Factor-*β* (TGF*β*) signaling [23, 24]. Regulation of tenocyte recruitment and the subsequent functional tendon regeneration appear to be directly regulated by Transforming Growth Factor-*β* (TGF*β*) signaling in the neonatal tissue [23].

In this preliminary investigation, we develop and characterize a decellularized tendon matrix (DTM) putty utilizing a novel enzymatic processing technique which promotes tenocyte maturation, preserves TGF*β* bioactivity, and promotes cell proliferation. Decellularized matrices have been utilized extensively in bone regeneration strategies to provide a biomimetic and bioactive substrate to support structural and biological healing. Tendon and dermal allografts are commercially available to support RCR, but their use has been limited due to the combination of unclear impact on clinical outcomes and limited evidence of bony ingrowth from the tuberosity into the allograft [25, 26]. Engineered decellularized matrices provide an opportunity to improve upon allograft technology by preserving the bioactivity of the tissue and generating a clinically more useful form factor for surgical application. We hypothesized that our novel method for creating a decellularized tendon matrix (DTM) putty would maintain bioactivity of the native tissue to enhance tendon-to-bone healing model.

## 2. Materials and Methods

### 2.1. Tendon Allograft and Tendon Processing

**Figure 1:**
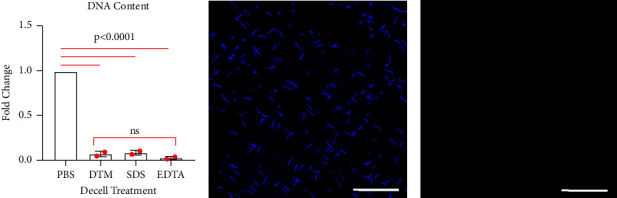
(a) DNA content was measured following decellularization using the using the DTM processing technique or using standard detergent methods, like 1% SDS and 0.1% EDTA. All decellularization methods effectively removed DNA content as compared to treatment with PBS (*p* < 0.0001; *n* = 2 donors) and no significant differences were found between decellularization treatments. Histological sections of (b) native tendon or (c) DTM decellularization stained with DAPI to identify cell nuclei, scale bar = 100 *µ*m. An ordinary one-way ANOVA was conducted to determine statistical significance in DNA content between the treatment groups, *F* (5, 6) = 515.1, *p* < 0.0001, and all further significance gained from Tukey's multiple comparison testing is listed on graph (a).

Achilles and patella tendon allografts were donated from Musculoskeletal Transplant Foundation (MTF, Edison, NJ) using the MTF Biologics Non-Transplantable Tissue Program. A total of 10 donors (5 males and 5 females with ages ranging from 18 to 61) were used in this study. Tendons were delivered on dry ice, thawed, and dissected into proximal, mid, and distal thirds for regional characterization (Figure 1(a)). The tendon was minced into smaller pieces and then decellularized using a tissue processing method involving DNase I at 50 U per 1 mL of 1X PBS in solution for 1 hour at 56°C with moderate shaking. Decellularized tendons were washed in excess PBS, frozen, and lyophilized until dry. DTM processing was compared to standard decellularization detergents sodium dodecyl sulfate (SDS, 1%) and ethylenediaminetetraacetic acid (EDTA, 0.1%) [27]. The untreated tendon was treated with PBS. DNA was isolated using DNeasy Blood and Tissue Kit per manufacturer's protocol (*n* = 2 donors/group). The DNA isolate was measured with Tecan's Nano Quant Plate analyzed using the 260/280 ratio read by Tecan's Infinite 200 Pro Plate reader. To develop an injectable tendon putty, 0.275 g of the lyophilized and decellularized tendon was placed in 1 mL of collagenase solution (collagenase type I at 2 mg/mL and collagenase type III at 1 mg/mL in 1X PBS) for enzymatic digestion. The tendons were enzymatically digested in this solution for 12 hours at 37°C with shaking and were then frozen and lyophilized. To remove collagenase solution from DTM, the tendons were reconstituted and placed in 100K molecular weight cutoff (MWCO) protein concentrators, spun at 8,000 × *g* for 5 minutes. Inactivation of the enzymatic digestion was verified using a Collagenase Activity Colorimetric Assay Kit, performed according to manufacturer's protocol. The reaction plate was quantified using Tecan's Infinite 200 Pro Plate reader at an absorbance of 345 nm. Collagenase activity was graphed as U/mg of the tendon (*n* = 3–6).

### 2.2. Protein Isolation, Quantification, and TGF*β* Protein Analysis

To obtain protein isolates, T-PER was added to the donor tendon samples with Protease Inhibitor Cocktail. The samples were homogenized using a tissue homogenizer and placed at 4°C for 2 hours to allow for protein extraction. The samples were filtered using 70 *μ*m cell strainers spinning at 2,000 × *g* for 10 minutes. Total protein was quantified using a disulfide reducing agent compatible microplate bicinchoninic acid assay (Micro-BCA) according to manufacturer's protocol. Protein concentrations of the experimental values were calculated using a linear model in micrograms of protein per milliliter of diluted sample. Protein isolates were used to quantify TGF*β* using TGF*β* Magnetic Bead 3 Plex Kit. A Luminex 200™ Instrument System was used to detect the analytes according to the Bead Panel's manufacturer protocol. 30 mg of total protein was placed within each of the wells for TGF*β* analysis, and the final output is expressed in picograms of TGF*β* per mL of sample.

### 2.3. Cell Proliferation Assay

**Figure 2:**
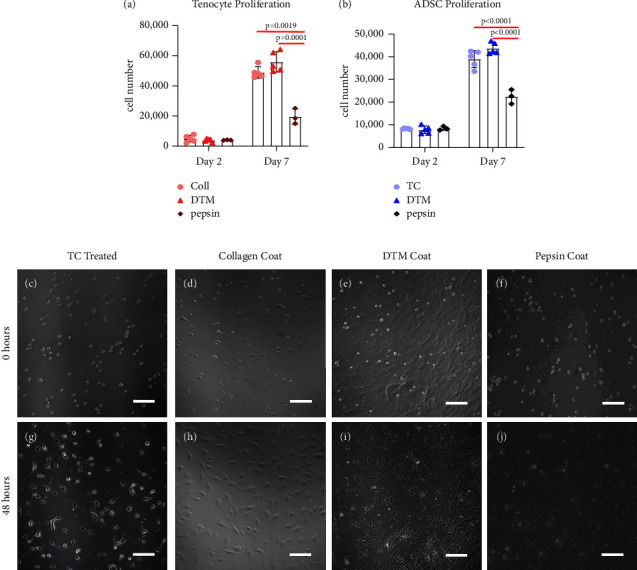
Primary tenocytes and ADSCs were plated at 20,000 cells/well and proliferation was quantified at 48 hours and 7 days (a, b) after plating, generating significantly different proliferation rates (*n* = 3–5 donors tested/group). DTM showed significantly more cell proliferation at day 7 than the pepsin groups in both tenocytes (*p* = 0.0001) and ADSCs (*p* < 0.0001). (c–j) Collagen coating, tissue-culture (TC) treated, DTM, and pepsin-coated plates were seeded with tenocytes and still images from live cell imaging were taken at 0 (c–f) and 48 hours (g–j) where variance in tenocyte cell morphology can be viewed. A two-way ANOVA was conducted revealing a statistically significant interaction between days and treatment groups on cell number for ADSCs, *F* (4, 18) = 31.77, *p* < 0.0001, and for tenocytes, *F* (4, 16) = 5.150, *p* = 0.0073. Statistically significant results from Tukey's multiple comparison test are reported on graphs (a) and (b). Scale bars = 100 *μ*m.

Human tenocytes, purchased from ZEN-BIO, and human adipose-derived stem cells (ADSCs), donated from CellTex, were plated onto the coated wells at 20,000 cells/well in DMEM/F12, 10% FBS, and 1% penicillin/streptomycin. All well plates were coated using 0.03 g/1 mL in 1X PBS of DTM or pepsin-based tendon products using 6 well tissue culture plates (*n* = 3–5 donors). Standard culture conditions were used as the control for both tenocytes and ADSCs (collagen-coated and tissue-culture treated, respectively). Collagen coating was done using collagen I at a concentration of 0.1 mg/1 mL in aqueous acetic acid. Plates were left to dry for 4 hours in a ventilated biosafety cabinet before the solution was removed and plates were sterilized under UV for 15 minutes. Proliferation was measured at 2 and 7 days using the Presto Blue Cell Viability Reagent according to manufacturer's protocol. Cell number was determined by relating absorbance values back to a standard curve of tenocytes and ADSCs, respectively. Cells were then washed with PBS and placed in cell lysis buffer for subsequent RNA isolation. To visualize the cell response to coatings, a live cell tissue imager was utilized using Nikon Eclipse Ti microscope, Andor Zyla sCMOS camera, and Oko Lab CO_2_/O_2_ plate chamber, powered by a Peka Light Engine. Over the course of 48 hours, 2,880 images were taken, with the first and last images being the ones displayed in Figure 2.

### 2.4. Differentiation Assay

RNA was isolated from the plates using Trizol Lysis Reagent according to manufacturer's protocol. The RNA concentration was measured using the Nano Quant Plate in a Tecan Infinite M200 Pro Plate reader. cDNA was reverse transcribed from 250 ng of RNA using qScript cDNA SuperMix on the ProFlex PCR System according to manufacturer's protocol. Quantitative reverse transcription polymerase chain reaction (qRT-PCR) was performed on a StepOnePlus Real-Time PCR System to measure expression of *Scleraxis (Scx)*, *Tenomodulin (Tnmd), Smad3*, and *Sox9* [28]. *Scx*, *Tnmd, Smad3*, and *Sox9* gene expressions were normalized to the housekeeping gene glyceraldehyde 3-phosphate dehydrogenase (GAPDH). SYBR Green Master Mix was utilized to detect amplicons, and PCR heat cycles were done according to SYBR Green manufacturer's protocol. Statistics and graphs were calculated using 2^(−∆Ct)^ [29]. Primer sequences are listed in Table 1.

### 2.5. Rheometry

Mechanical properties of DTM at different reconstitution levels were measured by rheological testing performed at the Centre for Industrial Technology out of Hampshire, United Kingdom. DTM was reconstituted at 1 mL, 3 mL, 5 mL, and 7 mL per 1 gram of human DTM, or 3 mL per 1 gram of rabbit DTM (*n* = 1 donor tested, 2-3 runs each concentration). Testing was performed on a research rheometer (DHR2, TA Instruments) fitted with a 20 mm scribed plate measuring system, with test gap set to 1100 *μ*m. All testing was done at 37°C, and a solvent trap cover was used to minimize drying of the exposed sample. Following a 60 second equilibration time at 37°C, the samples were exposed to oscillatory frequency sweeps from 100 rad/sec to 0.1 rad/sec. Sweeps were logarithmically scaled with 0.1% oscillation strain applied and 4 points per decade of frequency were collected. Immediately following the oscillation frequency sweep, the samples were exposed to an oscillatory stress sweep ranging from 1 to 100,000 Pa, 1 Hz oscillation frequency. A step termination was set such that if at any point the oscillation strain exceeded 1,500%, the test would immediately end. All analyses were performed in duplicate, both immediately after preparation and 30 minutes following preparation. Yield stress values were quantified by fitting an onset model to the complex modulus data.

### 2.6. Supraspinatus Tendon Repair in a Rabbit Model

All preclinical studies were performed at Colorado State University (CSU) by trained orthopaedic surgeons with institutional animal care and use committee (IACUC) approval. Eight healthy female New Zealand White rabbits (3–3.5 kg, 28 weeks old at time of initial tear) were used for this 14-week study. Rabbits received the tear at 28 weeks old to ensure the rabbits had reached bone maturity. Nine rabbits received the tear surgery; however, one rabbit died before the repair due to unrelated study circumstances. Rabbits were anaesthetized by subcutaneous injection of ketamine/acepromazine, shaved, and sterilized. A 2-3 cm skin incision was made, the supraspinatus tendon was detached from the greater tuberosity, and a Penrose drain was inserted into the free end of the tendon to prevent its spontaneous reattachment. The fascia was closed using a 3-0 absorbable Vicryl sutures, and the wound was closed with 4-0 sutures. The rabbits were allowed regular activity for 6 weeks to develop a chronic tendon tear model. After 6 weeks, the rabbits underwent tendon repair surgery. Using the same anesthesia and sterilization techniques as previously stated, the Penrose was identified and removed, and the supraspinatus tendon was fixed to the footprint at the greater tuberosity through 2 transosseous tunnels. The preparation of the greater tuberosity footprint was made by cleaning the remnant soft tendon tissues and trimming the superficial cortical bone to have a bleeding subchondral bone. High-strength sutures (#2-0 FiberWire, Arthrex, Naples, FL, USA) were passed through the transosseous tunnels and supraspinatus tendon and then tied in a standard fashion. After tendon repair surgery, one group received rabbit DTM (1 gram), processed according to the previously described DTM protocol, placed under the supraspinatus tendon near the osteotendinous junction (*n* = 4). The control animals received only the repair procedure (*n* = 4). Randomization of treatment was done on Microsoft Excel using the = RAND() function with blinded animal numbers prior to repair. Animals were euthanized 8 weeks after the repair procedure. The entire humeri and scapulae with their supraspinatus muscle and tendons were harvested for histological analysis. Contralateral shoulders were also collected.

### 2.7. Histological Analysis

Native Achilles and patella tendons, and processed DTM, were cut to a 1 cm × 1 cm square and cryo-embedded with Neg-50. Sections were cut to 6 *µ*m and then stained with Fluroshield Mounting Media with DAPI to confirm decellularization (*n* = 3). Rabbit shoulders were fixed for 2 days in 4% paraformaldehyde and decalcified for 4 weeks, shaking at 4°C in 19% EDTA solution, changing solution every other day. Shoulders were cut down and processed for paraffin embedding using a Tissue-Tek VIP 6 AI Vacuum Infiltration Processor, set to a 1-hour cycle time. Once the bones were embedded in paraffin, sections were cut to 6–8 *µ*m. Hall Brundt's Quadruple (HBQ) staining and H&E staining of rabbit shoulders were done to visualize cartilage and bone. HBQ stain is a histological technique which can distinguish between cartilage (blue) and bone (red) and was first published in 1986 [30]. HBQ uses direct red 81 stain to mark ossein and collagen fibers. Alcian blue stains structures involving connective tissue mucins and mucopolysaccharides [31]. VitroView™ Picrosirius Red Stain Kit was used according to manufacturer's instructions to view the orientation of collagen fibrils, and was imaged under brightfield microscopy.

### 2.8. Statistical Analysis

All data was plotted with and all statistical analysis was performed using Graph Pad Prism 8. Data were plotted so that each donor/sample represents a single dot on each graph in which the central line indicates the mean with error bars representing standard deviation. Statistical difference was determined by ANOVA, Tukey's post hoc comparison, and unpaired or paired *t*-tests. Tendon donor sample size was determined by the number of applicable and available research tissue. Animal sample size was determined by CSU cage availability and study funding.

An overview of catalog number and vendor information where key biological resources were purchased can be found in Supplemental Table 1.

## 3. Results

### 3.1. Allograft Sourcing for Decellularized Tendon Matrix (DTM)

**Figure 3:**
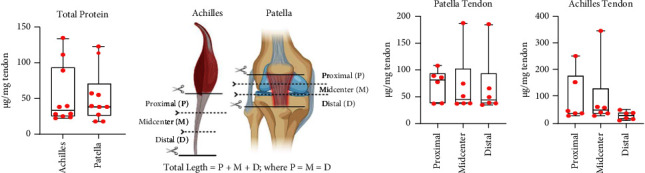
(a) Total protein content between native Achilles and patella tendons was analyzed and no significant difference was found between the tendon types (*p* = 0.7413; *n* = 10 donors). (b) Schematic diagram of Achilles and patella tendons dissected into equal thirds comprised of proximal (P), midcenter (M), and distal (D) regions. (c, d) The patellar and Achilles tendons had no significance in total protein content between proximal, mid, and distal regions (*p* = 0.9893 and *p* = 0.368, respectively; *n* = 6 donors). Ordinary one-way ANOVA was conducted to determine statistical significance in total protein content and treatment groups, *F* (2, 33) = 0.934, *p* = 0.403, in patella tendon protein content and treatment groups, *F* (2, 15) = 0.011, *p* = 0.989, and in Achilles tendon protein content and treatment groups*, F* (2, 15) = 1.069, *p* = 0.368.

Achilles and patellar tendons are the most readily available, abundant, and accessible allograft tendon tissues. It was unclear whether the protein content is different between these two tendon sources or whether sexual dimorphism is significant. No significant difference was observed in total protein content between Achilles and patellar tendons (Figure 3(a)) or across sex (Supplemental Figure 1). Since the vascularity of tendons decreases in the central region, in what is often described as the “watershed zone,” we aimed to further understand if protein content varied by location within the tendon. Achilles and patellar allografts were grossly dissected into proximal, midcenter, and distal segments based on dividing the total tendon length into thirds (Figure 3(b)). No significant difference in protein content was noted between proximal, midcenter, and distal divisions within tendons (Figures 3(c) and 3(d)). On this basis, decellularization of the tendon was performed on the composite of the whole Achilles and patellar tendon moving forward.

### 3.2. Decellularization Step Effectively Removes Cellular Content

To develop a method for effective decellularization without loss of matrix proteins and bioactivity, we compared an enzymatic decellularization protocol for DTM to previously published detergent-based techniques that used 1% SDS or 0.1% EDTA for 24 hours [27, 32]. All decellularization techniques effectively removed DNA relative to PBS (Figure 1(a)), with Tukey's HSD multiple comparison post hoc testing finding no significant difference between DNA content following decellularization using the DTM process compared to SDS or EDTA (Figure 1(a)). Removal of DNA was confirmed histologically by sectioning and staining with DAPI (Figures 1(b) and 1(c)).

### 3.3. Tendon Processing Maintains Protein Content and Creates a Tendon Putty

**Figure 4:**
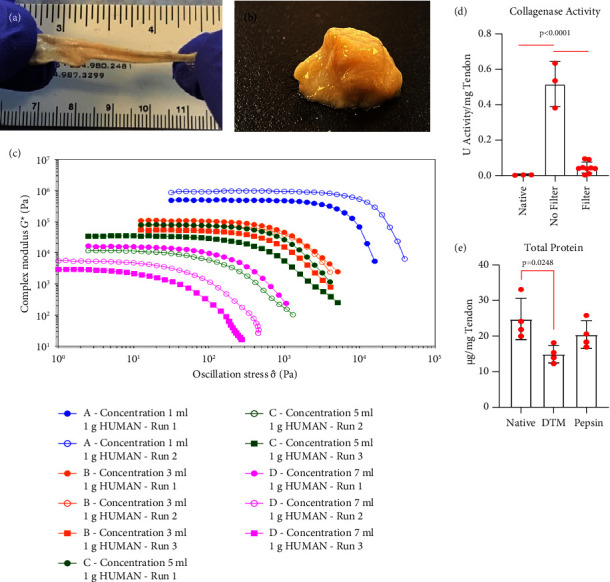
(a) DTM reconstituted and stretched to demonstrate the viscoelasticity of the DTM. (b) DTM reconstituted. (c) Oscillation stress sweep of human DTM with concentrations of 0, 1, 3, 5, and 7 g of lyophilized DTM to 1 mL of PBS resulted in all reconstitution concentrations tested to maintain an elastic modulus (*n* = 1 donor, 2-3 runs each). (d) Collagenase activity was measured to confirm successful removal of the added collagenase solution during enzymatic digestion. No significant differences were observed in collagenase activity between native tendon and enzymatically digested and filtered samples (*p* = 0.922; *n* = 3–10 donors). (e) The total protein of the samples was tested to confirm bioactivity was maintained (*n* = 4 donors) and no significant differences were found between native, DTM, and pepsin samples. An ordinary one-way ANOVA was conducted to determine statistical significance in collagenase activity and treatment groups, *F* (4, 22) = 18.06, *p* < 0.0001, and in total protein content between groups, *F* (2, 9) = 5.282, *p* = 0.0304. Additional significance from Tukey's multiple comparison is listed on graphs (d) and (e).

To enhance the surgical usability of DTM, we next enzymatically digested the decellularized tendon allograft using collagenase to break up the dense matrix and create a moldable form factor. The optimized digestion protocol for DTM generated a viscoelastic putty that can be stretched and reformed but maintained structural integrity (Figures 4(a) and 4(b)). Rheological properties of the DTM product were measured across various reconstitution concentrations (1 gram of tendon diluted in 1, 3, 5, or 7 mL PBS) to determine sample rigidity and structure strength. The oscillation stress sweeps reveal that samples at all dilutions display elastic dominant behavior across the full range of frequencies applied with overlapping mechanical properties at 3 and 5 mL dilutions (Figure 4(c)). The complex modulus plateaus (sample rigidity) and yield stress (sample strength) values decreased with increased dilution (Table 2). Putty-like gross structural properties were maintained in dilutions of 1 gram in 1–5 mL PBS; however, 7 mL exceeded the saturation limit of the tissue putty. Since these mechanical properties were achieved by enzymatically digesting the decellularized tendon allograft, we next aimed to ensure that enzymes were sufficiently removed to stop the digestion reaction. We found filtration of the digested allograft was sufficient to remove the collagenase (Figure 4(d)). We further show no significant differences in the total protein content using our collagenase protocol to digest the tendon allograft and a previously published pepsin digestion protocol (Figure 4(e)) [30, 33].

### 3.4. DTM Promotes Cell Proliferation over Pepsin-Digested Product

Bioactivity of the DTM was characterized *in vitro* using cell proliferation and gene expression analysis. Cellular proliferation was measured using the Presto Blue Assay for both tenocytes and adipose-derived stem cells (ADSCs). No significant differences between treatment groups were found 48 hours after treatment in both tenocytes and ADSCs. However, 7 days following the initial seeding, DTM-coated plates had significantly more tenocytes and ADSCs compared to the pepsin-coated plates, respectively (Figures 2(a) and 2(b)). While DTM-coated plates were not significantly higher than the controls, the DTM-coated plates trended higher than the controls for both tenocytes and ADSCs at day 7. Still images taken at the time of cell plating and 48 hours later show that tenocytes have a varied cell morphology when cultured on different coatings. Specifically, tenocytes were found to have a more native-like cell morphology on DTM-coated plates (Figure 2(i)) as compared to the standard tissue culture plate (Figure 2(g)), collagen-coated plate (Figure 2(h)) and pepsin-coated plate (Figure 2(j)). Time lapsed videos show the increased proliferation (Figures 2(k) and 2(l)) on the DTM coating that was quantified by Presto Blue (Figures 2(a) and 2(b)).

### 3.5. DTM Promotes Tenocyte Maturation through Maintenance of TGF*β* Bioactivity

**Figure 5:**
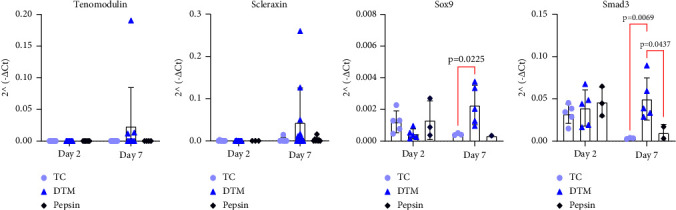
RNA isolated from ADSCs cultured on tissue-culture (TC) treated, DTM, and pepsin-coated plates was measured for level of tenocyte differentiation by probing for tenocyte markers (*n* = *3–5 donors*) (a) *Tenomodulin* (*Tnmd*) and (b) *Scleraxis* (*Scx*), (c) tenocyte recruiter *Sox9*, and (d) TGF-*β* marker *Smad3*. DTM had significantly more *Sox9* (*p* = 0.023) and *Smad3* (*p* = 0.0395) than the control at day 7. Performing a two-way ANOVA revealed a statistically significant interaction between days and treatment groups on Sox9 values, *F* (2, 16) = 6.365, *p* = 0.0093, and on Smad3 values, *F* (2, 17) = 3.907, *p* = 0.0402. Statistically significant results from Tukey's multiple comparison test are reported for Sox9 and for Smad3 on each respective graph above. Two-way ANOVA for *Scx*, *F* (2, 23) = 0.4200, *p* = 0.6620, and *Tnmd*, *F* (2, 19) = 0.9607, *p* = 0.4005, was found to have no statistical significance.

To characterize stem cell differentiation of ADSC in response to the DTM, RNA was harvested at day 2 and 7 following seeding. Expressions of the tenogenic markers *Tenomodulin* (*Tnmd*) and *Scleraxis* (*Scx*), and the chondrogenic transcription factor SRY-box transcription factor 9 (*Sox9*), were measured using qRT-PCR. *Tnmd* expression trended higher for ADSCs when cultured on DTM-coated plates as compared to pepsin groups and the control conditions (Figure 5(a)) at day 7. *Scx* expression also trended higher in the DTM-coated plates at day 7 as compared to both pepsin and control (Figure 5(b)). In addition to promoting tendon differentiation, DTM promoted chondrogenic differentiation as evident by an increase in *Sox9* expression at day 7 than control (Figure 5(c)). Since soluble growth factors were not provided and TGF*β* has been implicated in the development of the fibrocartilage enthesis [23, 24], we next looked to see if *Smad3,* the downstream effector of TGF*β*, was upregulated by the ADSCs following culture on DTM. DTM resulted in significantly more *Smad3* expression at day 7 compared to control culture (Figure 5(d)).

**Figure 6:**
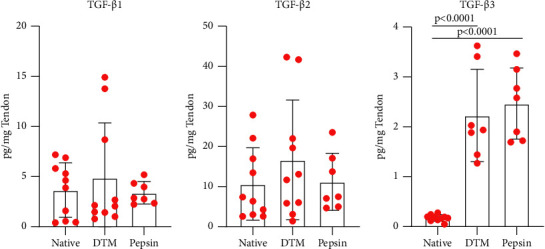
The TGF*β* profile was then measured to determine bioactivity of the processed tendons compared to the native tendons (*n* = 10 donors). No statistical significance was found in (a) TGF*β*1 and in (b) TGF*β*2 between combined native tendons, DTM, and pepsin. However, statistical significance was found in (c) TGF*β*3 between the combined native tendons and DTM (*p* < 0.0001) and combined native tendon and pepsin (*p* < 0.0001), with no statistical difference between DTM and pepsin. One-way ANOVA was conducted to determine significance between treatment groups on TGF-*β*1, *F* (2, 24) = 4.054, *p* = 0.6712, on TGF-*β*2, *F* (2, 24) = 0.8386, *p* = 0.4446, and on TGF-*β*3, *F* (2, 21) = 35.55, *p* < 0.0001. Statistically significant results from Tukey's multiple comparison test are reported on graph (c).

Based on this increased expression of *Smad3*, we next looked to see whether the tendon allografts contained measurable levels of TGF*β* at a protein level using Multiplex assays for TGF*β* I, II, and III. Importantly, we found that adult tendon allografts do contain TGF*β* and that there was no significant decrease in TGF*β* I (Figure 6(a)) or TGF*β* II (Figure 6(b)) expression following decellularization and enzymatic digestion. Interestingly, we did find significantly more TGF*β* III (Figure 6(c)) in digested samples, DTM, and pepsin, compared to native tendon allografts. Additionally, no significant differences were found between male and female donors in patella or Achilles tendons (Sup.  Figure 1B). Further, we tested differences in TGF*β* activity between male and female donors of the combined tendon samples. No significant differences were found between sexes in TGF*β*I (Sup.  Figure 2A), TGF*β*II (Sup.  Figure 2B), or TGF*β*III (Sup.  Figure 2C). Additionally, no correlations were found between TGF-*β* I, II, or III activity and age of the donor (Sup. Figures 2A–2C).

### 3.6. DTM Augments Rotator Cuff Repair by Improving Enthesis Biology

**Figure 7:**
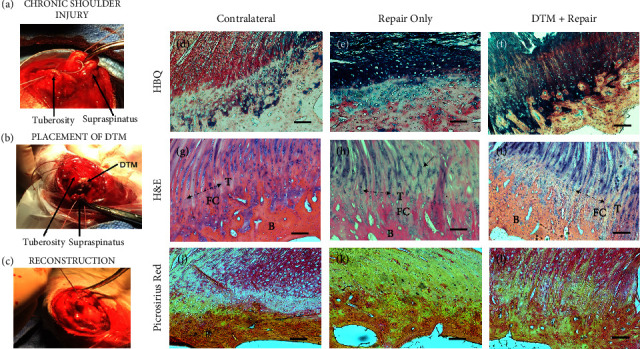
Rotator cuff repair (RCR) surgery was performed in a rabbit model with a chronic rotator cuff tear (*n* = 4/group). (a) Initial rotator cuff tear of the supraspinatus; (b) DTM placed under the supraspinatus 6 weeks after the initial tear; (c) repair of the initial tear, suture-anchoring the supraspinatus to the greater tuberosity. 8 weeks following the repair, shoulders were harvested, processed through histological analysis, and stained with (d–f) HBQ, (g–i) H&E, and (j–l) Picrosirius Red. Images display the bone, fibrocartilage, and tendon tissues at the tendon enthesis in (d, g, j) contralateral shoulders, (e, h, k) repair only, and (f, i, l) DTM treatment with repair. T = tidemark; FC = fibrocartilaginous zone; B = bone; arrow in (h) shows collagen fiber orientation. Scale bars represent 100 *μ*m.

To determine if the *in vitro* bioactivity translated to a regenerative response, we completed an *in vivo* study in New Zealand White rabbits. Females were chosen due to the lack of sexual variability found within our preliminary data. Importantly, no significant differences were found between protein content of male and female Achilles or patella tendons (Supplemental Figure 1B) and additionally no differences were found in TGF*β*I, II, and III content between male and female tendons (Supplemental Figures 2A–2C). Because many RCR failures are associated with chronic injuries, we utilized a rabbit model with a chronic rotator cuff tear (Figure 7(a)). In the groups that received DTM, the putty (1 mg rabbit DTM diluted in 3 mL PBS, rheological properties of rabbit DTM can be found in Supplemental Figure 3) was molded directly onto the greater tuberosity (Figure 7(b)). Repair then proceeded by securing the supraspinatus tendon to the greater tuberosity through 2 transosseous tunnels (Figure 7(c)). HBQ staining at the tendon-bone interface shows morphological differences between DTM (Figure 7(f)) and the repair-only control group (Figure 7(e)). Images of contralateral shoulder enthesis reveal a cartilage transition, indicated by the red and blue colocalized staining in the matrix (Figure 7(d)). DTM also showed a cartilage transition at the junction (Figure 7(f)), yet little to no cartilage transition zone was present in the repair only (Figure 7(e)). These zones are labeled as FC = fibrocartilage zone, T = tidemark, and B = bone. Additionally, H&E staining reveals disoriented collagen fibrils near the enthesis within the RCR group alone (Figure 7(h), labeled with the arrow) as compared to both the contralateral shoulder and the DTM with repair groups. To further view the collagen orientation, we stained with Picrosirius Red showing more similarities in stain between the DTM group (Figure 7(l)) and the contralateral shoulder as compared to the repair only group (Figure 7(j)). Additional histological images can be found in Supplemental Figure 4.

## 4. Discussion

This study tested bioactivity of our engineered DTM putty with the long-term goal of promoting tendon regeneration during RCR. Because most rotator cuff tears result in diminished functionality of the shoulder joint, surgical repair remains the gold standard treatment for full thickness rotator cuff tears that appear to be amenable to repair. RCRs continue to have high clinical failure rates, with the majority of failures occurring at the tendon-bone interface due to the formation of a mechanically inferior scar tissue [5, 12, 34]. Given the importance of tendon healing for proper success of RCR, there remains a clinical need to find new, readily translatable approaches to reduce fibrosis and promote regeneration of a more native enthesis structure.

Currently, there are few products designed to augment RCR, and those with clinical efficacy studies have reported minimal benefit [35]. Recent tendon allograft studies suggest that modern surgical techniques yield functional improvements, yet MRI images illustrate poor tissue regeneration with tendon allograft and structural failure rate of 57% [10, 36]. Large clinical studies on RCR with various types of allograft are lacking, and there are no studies showing strong evidence of tissue regrowth into the allograft or regeneration of the enthesis structure [26]. As an alternative to tendon allografts, dermal, small intestine submucosa and fascia lata-derived human allografts have been tested for rotator cuff repair [37–39]. While these allografts lack the standalone biomechanical properties of tendons [40], there are some clinical data suggesting efficacy [41–45]. Gupta et al. [42] performed a prospective observation of 24 patients who underwent a mini-open massive rotator cuff repair using a human dermal allograft at average follow-up of 3 years and demonstrated significant improvements in pain (VAS score; *p* = 0.0002), range of motion (forward flexion, external rotation, shoulder abduction; *p* ≤ 0.001), and patient reported outcomes (ASES, SF-12; *p* ≤ 0.3). At final follow-up, ultrasonographic evaluation revealed no evidence of complete tears, but 24% of patients only had a “partially intact” repair. In a more recent retrospective study in 12 patients (13 shoulders, 1 bilateral) that underwent revision rotator cuff repair with human acellular dermal allograft augmentation, Petri et al. found no complications, adverse reactions to the patch, or need for further surgery. Additionally, mean satisfaction was 9/10 (range, 2–10) and the total ASES score improved by 21.3 at mean follow-up of 2.5 years [46]. Notably, only the functional component of the ASES score improved significantly (*p* < 0.05) and neither the total ASES score nor the pain component score improved by statistically significant amounts despite improving trends.

In this study, we have developed a processing technique to form a decellularized tendon allograft (DTM) putty with an optimized form factor to match modern arthroscopic surgical RCR techniques [32, 47]. Decellularization is considered a critical step in preventing a host inflammatory response to the allograft and disease transmission [48, 49]. Unlike traditional tendon allograft processing techniques, we remove cellular content without detergents in an effort to maintain the protein bioactivity native within the tendon matrix, prevent harsh soft tissue matrix degradation, and reduce long processing time seen with detergents [27, 32, 33, 50].

To improve the form factor of the allograft so that it was moldable for surgical application, we also added a collagenase digestion step. Conventional methods to circumvent allograft's poor form factor include enzymatic digestion or solubilization of the decellularized tissue [33, 51, 52]. Traditional solubilization techniques use pepsin to preserve the ECM composition and structure. Pepsin is a proteolytic enzyme targeting telopeptide bonds of collagen giving rise to collagen fibril aggregates [52]. Yet, pepsin necessitates an acidic base to facilitate the self-assembly of collagen peptides after being neutralized to physiologic pH, and this certainly alters the structural and functional integrity of the matrix [52]. Farnebo et al. generated an injectable decellularized hydrogel derived from human flexor tendon using a pepsin solubilization technique to augment various tendonopathies and tendon injuries [33]. When testing their product in a murine Achilles injury model, the groups that received the hydrogel resulted in higher ultimate failure load at earlier healing stages as compared to the control groups [53]. While this shows promising results of an injectable-based decellularized tendon construct to augment Achilles tendon injuries, a more moldable and less viscous form would be more suitable to treat rotator cuff repairs.

In testing DTM *in vitro* using tenocytes and ADSCs, we demonstrate greater cell proliferation in both cell types compared to pepsin-based tendon products. Gene analysis also suggested that DTM promoted the expression of *Scx* and *Tnmd*, tendon-specific transcription factors required for tendon development, proliferation, and maturation [18, 54, 55]. Furthermore, ADSCs cultured with DTM showed more *Sox9* expression than all other groups tested, suggesting that DTM drives the *Scx*^+^/*Sox9*^+^ phenotype seen in tendon progenitor cells, the cell type required for postnatal tendon repair [21, 22].

TGF*β* has been reported to be critical for scarless tendon healing in neonatal mice through the recruitment and proliferation of *Scx*^*+*^/*Sox9*^*+*^ cells [13, 15, 23, 24, 56]. In our study, we found that ADSCs cultured on DTM significantly upregulated *Smad3* expression, suggesting that DTM promotes the *Scx*^*+*^/*Sox9*^*+*^ phenotype through retention of TGF*β*. Consequently, we next measured the amount of TGF*β* within the DTM samples and found that our processing technique did not lead to a significant loss of TGF*β* when compared to the native tendon. While several studies have shown that the upregulation of TGF*β*I activity is associated with the age-related degenerative pathology in rotator cuff muscles, other studies display the necessary role TGF*β*I has during tendon enthesis repair [57]. Importantly, TGF*β*I was found to be a key player throughout the early phases of rotator cuff healing *in vivo* coinciding with early ECM synthesis [58–60]. In fact, it was determined to be the delayed upregulation of TGF*β*I (seen 8 weeks following injury) that was correlated with scarring [34, 59, 61]. Additionally, various cell-ECM interactions such as substrate elasticity and proteolyticdegradation of ECM macromolecules influence TGF*β* levels [62–67]. This suggests that DTM bioactivity may be due to the retention of the TGF*β* family of proteins, which consequently recruit* Scx*^+^/*Sox9*^+^ tendon progenitor cells known to stimulate repair at the enthesis.

Preclinical animal studies are a critical step in demonstrating the efficacy of novel technologies for tendon repair. Due to limited applicability of rodent models for RCR in humans, large animal models, including rabbit and sheep, are typically required to explore efficacy and serve as more translationally relevant models [68–71]. Importantly, chronic injury rabbit models show muscle atrophy, fatty infiltration, and migration of the subscapularis tendon underneath a bony arch, characteristics comparable to massive tears in humans [69, 72]. For these reasons, we performed a pilot study in a rabbit model with a chronic rotator cuff injury, previously shown to replicate pathological changes consistent with a massive rotator cuff injury [73, 74]. The rabbit model is also sufficiently sized to enabled use of advanced arthroscopic surgical approaches used today to reduce re-tear rates [75–79].

Quality of the chronic rotator cuff injury repair in the DTM group was compared to standard surgical repair without biological augmentation after 8 weeks. When elucidating the pathogenesis of rotator cuff tears, Gigante et al. consistently found round cells characteristic of a chondrogenic lineage in rotator cuff tears, as compared to calcified fibrocartilage typically seen in non-torn tendon enthesis [80]. Our results presented similar findings as the RCR only group was shown to have cells phenotypically congruent with chondrocytes lacking the highly aligned calcified fibrocartilage region of a normal enthesis. In contrast, the DTM repair group showed longitudinally oriented collagen fibers forming at the enthesis with a zone of calcified cartilage at the bone-to-tendon interface that was more similar to the non-repaired contralateral shoulder control. Failure of RCR in chronic/massive tears has specifically been associated with the formation of mechanically weak fibrovascular scars lacking the zone of calcified cartilage [14–16]. These preliminary *in vivo* data justify additional studies to further define and quantify the four distinct regions of fibrocartilaginous entheses, especially the uncalcified and calcified zones of fibrocartilage.

This study has some limitations. The first limitation was the small *n* value of the rabbit model enabling only preliminary data. We chose to use the rabbit model for these pilot study studies as it is a more clinically relevant method for rotator cuff repair; however, the expense and time of this model limited the *n* value. Additionally, these promising results from the pilot study performed necessitate more future *in vivo* studies to further confirm and expand upon these results, including biomechanical studies and quantitative histomorphometry. Another limitation concerning the *in vivo* study design was not testing the efficacy of pepsin-digested tendon in our rabbit model. Adding this as an experimental group to future studies in comparison to DTM would further expand upon the reported *in vitro* results. Limitations involving the *in vitro* studies include the difference in concentrations of well plate coatings between standard collagen-coated plates, DTM, and pepsin. The last limitation was not testing rabbit DTM for metabolic activity of tenocytes and ADSCs *in vitro.* Testing the human DTM in an animal model will be considered in future steps although we also must consider possible xenographic immune reactions.

DTM represents an adaptation to allograft processing that can maintain bioactivity to promote better tissue regeneration. By utilizing tendon allografts as the basis for the DTM, we aimed to maintain the tissue-specific bioactivity of the tendon. A similar approach has been used to develop an injectable decellularized tendon hydrogel; however, efficacy was only tested in Achilles injury models [27]. While injectable systems may offer advantages, such as non-surgical implantation, the disadvantages of injectable systems consist of highly digested matrices, low tendon material concentrations per injection, and the possibility of the product moving. However, DTM can be applied arthroscopically during RCR which circumvents the need for an injectable-based platform.

Complex strategies for engineered decellularized matrices have been proposed as a regenerative material, aiming to incorporate key parts of the tendon matrix and add back in cells or growth factors to promote cell migration or differentiation [9, 81–83]. These approaches may be very promising in the future but can be challenging and long to translate to the clinic.

## Figures and Tables

**Table 1:** PCR primer sequences.

Marker	Forward Sequence	Reverse Sequence
Scleraxis *(Scx)*	CGAGAACACCCAAGCCCAAAC	CTCCGAATCGCAGTCTTTCTGTC
*Tenomodulin (Tnmd)*	TGGGTGGTCCCTCAAGTGAAAGT	CTCGACGGCAGTAAATACAACAATA
*Smad3*	*TGAGGCTGTCTACCAGTTGACC*	*GTGAGGACCTTGTCAAGCCACT*
*Sox9*	*GCTCAGCAAGACGCTGGGCA*	*CCGGAGGAGGAGTGTGGCGA*
*GAPDH*	TGACGCTGGGGCTGGCATTG	GGCTGGTGGTCCAGGGGTCT

**Table 2:** Summary of rheological properties of DTM, with the reconstitution concentration in column one, followed by complex modulus plateau (Pa), phase angle (^o^), and yield stress (Pa) (*n* = 1 donor, run 1 time).

Group	Concentration	Complex modulus plateau (Pa)	Phase angle plateau (^o^)	Yield stress (Pa)
A	1 mL/1 g DTM	929000	11.3	12200
B	3 mL/1 g DTM	50500	15.2	683
C	5 mL/1 g DTM	32300	15.2	404
D	7 mL/1 g DTM	2640	18	76

## Data Availability

The data used to support the findings of this study are available from the corresponding author upon request.

## Supplementary Materials

Supplementary Materials Supplemental Figure 1: (A) male and female donors (*n* = 5 donors each) on the *x*-axis displayed by their respective age on the *y*-axis; (B) no significance was found in total protein between patella and Achilles tendons of male and female donors. Kruskal–Wallis test was performed to measure differences in protein content between male and female tendon types, H(3) = 0.5086, *p* = 0.917. Supplemental Figure 2: Mann–Whitney tests were performed to find no sex-specific significant differences in (A) TGF-*β*1, *U* = 12, *p* > 0.999, (B) TGF-*β*2, *U* = 12, *p* > 0.999, or (C) TGF-*β*3, *U* = 12, *p* > 0.999, for the male (*n* = 5 donors) and female (*n* = 5 donors) donors. (D–F) Additionally, there were no correlations found between age and TGF-*β* I, II, or III activity. Supplemental Figure 3: Rabbit DTM was tested for (A) total protein content, (B–D) TGF*β* activity, and (E) rheological analysis prior to performing *in vivo* studies. (A–D) Rabbit DTM had significantly more protein content than native rabbit tendon (*p* = 0.0033). Significantly more TGF*β* activity was determined through unpaired two-tailed *t*-tests (*p* = 0.0062; *p* < 0.0001; *p* < 0.0001, respectively; *n* = 7 rabbit donors). (E) Rabbit DTM maintained an elastic dominant behavior at all frequencies tested. Supplemental Figure 4: Additional histology images were provided to represent the variance in animals. HBQ images (A–C) were obtained to show collagen (red) and GAGs (blue) at the enthesis. Additional H&E stains (D–F) show differences in the fibrocartilaginous area within enthesis. Scale bars = 100 *μ*m. Supplementary Table 1: Overview of catalog number and vendor where key biological resources were purchased.

## References

[1] Yelin E., Weinstein S., King T. (2016). The burden of musculoskeletal diseases in the United States. *Seminars in Arthritis and Rheumatism*.

[2] ltd R., (n.d.) M. (2019). *Sports medicine market report suite-united states-2020–2026-medsuite*.

[3] Aguado G., Obando D. V., Herrera G. A., Ramirez A., Llinas P. J. (2019). Retears of the rotator cuff: an ultrasonographic assessment during the first postoperative year. *Orthopaedic Journal of Sports Medicine*.

[4] Collin P., Betz M., Herve A. (2020). Clinical and structural outcome 20 years after repair of massive rotator cuff tears. *Journal of Shoulder and Elbow Surgery*.

[5] Galatz L. M., Ball C. M., Teefey S. A., Middleton W. D., Yamaguchi K. (2004). The outcome and repair integrity of completely arthroscopically repaired large and massive rotator cuff tears. *The Journal of Bone and Joint Surgery*.

[6] Katthagen J. C., Bucci G., Moatshe G., Tahal D. S., Millett P. J. (2018). Improved outcomes with arthroscopic repair of partial-thickness rotator cuff tears: a systematic review. *Knee Surgery, Sports Traumatology, Arthroscopy*.

[7] Lafosse L., Brozska R., Toussaint B., Gobezie R. (2007). The outcome and structural integrity of arthroscopic rotator cuff repair with use of the double-row suture anchor technique. *The Journal of Bone and Joint Surgery*.

[8] Millett P. J., Horan M. P., Maland K. E., Hawkins R. J. (2011). Long-term survivorship and outcomes after surgical repair of full-thickness rotator cuff tears. *Journal of Shoulder and Elbow Surgery*.

[9] Rashid M. S., Cooper C., Cook J. (2017). Increasing age and tear size reduce rotator cuff repair healing rate at 1 year. *Acta Orthopaedica*.

[10] Zumstein M. A., Jost B., Hempel J., Hodler J., Gerber C. (2008). The clinical and structural long-term results of open repair of massive tears of the rotator cuff. *Journal of Bone and Joint Surgery American Volume*.

[11] McColl A. H., Lam P. H., Murrell G. A. C. (2019). Are we getting any better? A study on repair integrity in 1600 consecutive arthroscopic rotator cuff repairs. *JSES Open Access*.

[12] Kunze K. N., Rossi L. A., Beletsky A., Chahla J. (2020). Does the use of knotted versus knotless transosseous equivalent rotator cuff repair technique influence the incidence of retears? A systematic review. *Arthroscopy: The Journal of Arthroscopic and Related Surgery*.

[13] Benjamin M., Toumi H., Ralphs J. R., Bydder G., Best T. M., Milz S. (2006). Where tendons and ligaments meet bone: attachment sites (‘entheses’) in relation to exercise and/or mechanical load. *Journal of Anatomy*.

[14] Bunker D., Illie V., Illie V., Nicklin S. (2019). Tendon to bone healing and its implications for surgery. *Muscle Ligaments and Tendons Journal*.

[15] Gerber C., Schneeberger A. G., Perren S. M., Nyffeler R. W. (1999). Experimental rotator cuff repair. A preliminary study. *The Journal of Bone and Joint Surgery*.

[16] Kovacevic D., Rodeo S. A. (2008). Biological augmentation of rotator cuff tendon repair. *Clinical Orthopaedics and Related Research*.

[17] Galatz L. M., Gerstenfeld L., Heber-Katz E., Rodeo S. A. (2015). Tendon regeneration and scar formation: the concept of scarless healing. *Journal of Orthopaedic Research*.

[18] Howell K., Chien C., Bell R. (2017). Novel model of tendon regeneration reveals distinct cell mechanisms underlying regenerative and fibrotic tendon healing. *Scientific Reports*.

[19] Huang A. H., Lu H. H., Schweitzer R. (2015). Molecular regulation of tendon cell fate during development. *Journal of Orthopaedic Research*.

[20] Huang A. H., Watson S. S., Wang L. (2019). Requirement for scleraxis in the recruitment of mesenchymal progenitors during embryonic tendon elongation. *Development*.

[21] Ideo K., Tokunaga T., Shukunami C. (2020). Role of Scx+/Sox9+ cells as potential progenitor cells for postnatal supraspinatus enthesis formation and healing after injury in mice. *PLoS One*.

[22] Liu E. S., Martins J. S., Zhang W., Demay M. B. (2018). Molecular analysis of enthesopathy in a mouse model of hypophosphatemic rickets. *Development*.

[23] Kaji D. A., Howell K. L., Balic Z., Hubmacher D., Huang A. H. (2020). Tgf*β* signaling is required for tenocyte recruitment and functional neonatal tendon regeneration. *Elife*.

[24] Wang X., Xie L., Crane J. (2018). Aberrant TGF-beta activation in bone tendon insertion induces enthesopathy-like disease. *Journal of Clinical Investigation*.

[25] Moore M. S., McAuley J. P., Young A. M., Engh Sr C. A. (2006). Radiographic signs of osseointegration in porous-coated acetabular components. *Clinical Orthopaedics and Related Research*.

[26] Thangarajah T., Lambert S. (2015). Management of the unstable shoulder. *BMJ*.

[27] Farnebo S., Woon C. Y., Kim M., Pham H., Chang J. (2014). Reconstruction of the tendon-bone insertion with decellularized tendon-bone composite grafts: comparison with conventional repair. *The Journal of Hand Surgery*.

[28] Spang C., Chen J., Backman L. J. (2016). The tenocyte phenotype of human primary tendon cells in vitro is reduced by glucocorticoids. *BMC Musculoskeletal Disorders*.

[29] Schmittgen T. D., Livak K. J. (2008). Analyzing real-time PCR data by the comparative C(T) method. *Nature Protocols*.

[30] Hall B. (1986). The role of movement and tissue interactions in the development and growth of bone and secondary cartilage in the clavicle of the embryonic chick. *Development*.

[31] Lison L. (1954). Alcian blue 8 G with chlorantine fast red 5 B. A technic for selective staining of mucopolysaccharides. *Stain Technology*.

[32] Lovati A. B., Bottagisio M., Moretti M. (2016). Decellularized and engineered tendons as biological substitutes: a critical review. *Stem Cells International*.

[33] Farnebo S., Woon C. Y., Schmitt T. (2014b). Design and characterization of an injectable tendon hydrogel: a novel scaffold for guided tissue regeneration in the musculoskeletal system. *Tissue Engineering Part A*.

[34] Galatz L. M., Sandell L. J., Rothermich S. Y. (2006). Characteristics of the rat supraspinatus tendon during tendon-to-bone healing after acute injury. *Journal of Orthopaedic Research*.

[35] Docheva D., Muller S. A., Majewski M., Evans C. H. (2015). Biologics for tendon repair. *Advanced Drug Delivery Reviews*.

[36] Cho N. S., Yi J. W., Rhee Y. G. (2009). Arthroscopic biceps augmentation for avoiding undue tension in repair of massive rotator cuff tears. *Arthroscopy: The Journal of Arthroscopic and Related Surgery*.

[37] Aurora A., McCarron J., Iannotti J. P., Derwin K. (2007). Commercially available extracellular matrix materials for rotator cuff repairs: state of the art and future trends. *Journal of Shoulder and Elbow Surgery*.

[38] Longo U. G., Lamberti A., Maffulli N., Denaro V. (2010). Tendon augmentation grafts: a systematic review. *British Medical Bulletin*.

[39] Ricchetti E. T., Aurora A., Iannotti J. P., Derwin K. A. (2012). Scaffold devices for rotator cuff repair. *Journal of Shoulder and Elbow Surgery*.

[40] Youngstrom D. W., Barrett J. G. (2016). Engineering tendon: scaffolds, bioreactors, and models of regeneration. *Stem Cells International*.

[41] Bond J. L., Dopirak R. M., Higgins J., Burns J., Snyder S. J. (2008). Arthroscopic replacement of massive, irreparable rotator cuff tears using a GraftJacket allograft: technique and preliminary results. *Arthroscopy: The Journal of Arthroscopic and Related Surgery*.

[42] Gupta A. K., Hug K., Berkoff D. J. (2012). Dermal tissue allograft for the repair of massive irreparable rotator cuff tears. *The American Journal of Sports Medicine*.

[43] Gupta A. K., Hug K., Boggess B., Gavigan M., Toth A. P. (2013). Massive or 2-tendon rotator cuff tears in active patients with minimal glenohumeral arthritis: clinical and radiographic outcomes of reconstruction using dermal tissue matrix xenograft. *The American Journal of Sports Medicine*.

[44] Petri M., Greenspoon J. A., Moulton S. G., Millett P. J. (2016). Patch-augmented rotator cuff repair and superior capsule reconstruction. *The Open Orthopaedics Journal*.

[45] Wong I., Burns J., Snyder S. (2010). Arthroscopic GraftJacket repair of rotator cuff tears. *Journal of Shoulder and Elbow Surgery*.

[46] Petri M., Warth R. J., Horan M. P., Greenspoon J. A., Millett P. J. (2016). Outcomes after open revision repair of massive rotator cuff tears with biologic patch augmentation. *Arthroscopy: The Journal of Arthroscopic and Related Surgery*.

[47] Kryger G. S., Chong A. K., Costa M., Pham H., Bates S. J., Chang J. (2007). A comparison of tenocytes and mesenchymal stem cells for use in flexor tendon tissue engineering. *The Journal of Hand Surgery*.

[48] Gilbert T. W., Freund J. M., Badylak S. F. (2009). Quantification of DNA in biologic scaffold materials. *Journal of Surgical Research*.

[49] Thangarajah T., Pendegrass C. J., Shahbazi S., Lambert S., Alexander S., Blunn G. W. (2015). Augmentation of rotator cuff repair with soft tissue scaffolds. *Orthopaedic Journal of Sports Medicine*.

[50] Roth S. P., Erbe I., Burk J. (2018). Decellularization of large tendon specimens: combination of manually performed freeze-thaw cycles and detergent treatment. *Methods in Molecular Biology*.

[51] Ozasa Y., Amadio P. C., Thoreson A. R., An K. N., Zhao C. (2014). Repopulation of intrasynovial flexor tendon allograft with bone marrow stromal cells: an ex vivo model. *Tissue Engineering Part A*.

[52] Saldin L. T., Cramer M. C., Velankar S. S., White L. J., Badylak S. F. (2017). Extracellular matrix hydrogels from decellularized tissues: structure and function. *Acta Biomaterialia*.

[53] Kim M. Y., Farnebo S., Woon C. Y. L., Schmitt T., Pham H., Chang J. (2014). Augmentation of tendon healing with an injectable tendon hydrogel in a rat Achilles tendon model. *Plastic and Reconstructive Surgery*.

[54] Docheva D., Hunziker E. B., Fassler R., Brandau O. (2005). Tenomodulin is necessary for tenocyte proliferation and tendon maturation. *Molecular and Cellular Biology*.

[55] Shukunami C., Takimoto A., Nishizaki Y. (2018). Scleraxis is a transcriptional activator that regulates the expression of Tenomodulin, a marker of mature tenocytes and ligamentocytes. *Scientific Reports*.

[56] Zhou S., Eid K., Glowacki J. (2004). Cooperation between TGF-beta and Wnt pathways during chondrocyte and adipocyte differentiation of human marrow stromal cells. *Journal of Bone and Mineral Research*.

[57] Tazawa R., Uchida K., Kenmoku T. (2021). Increasing transforming growth factor-beta concentrations with age decrease apelin in the rat rotator cuff. *Journal of Orthopaedic Surgery and Research*.

[58] Kobayashi M., Itoi E., Minagawa H. (2006). Expression of growth factors in the early phase of supraspinatus tendon healing in rabbits. *Journal of Shoulder and Elbow Surgery*.

[59] Prabhath A., Vernekar V. N., Sanchez E., Laurencin C. T. (2018). Growth factor delivery strategies for rotator cuff repair and regeneration. *International Journal of Pharmaceutics*.

[60] Wurgler-Hauri C. C., Dourte L. M., Baradet T. C., Williams G. R., Soslowsky L. J. (2007). Temporal expression of 8 growth factors in tendon-to-bone healing in a rat supraspinatus model. *Journal of Shoulder and Elbow Surgery*.

[61] Dahlgren L. A., Mohammed H. O., Nixon A. J. (2005). Temporal expression of growth factors and matrix molecules in healing tendon lesions. *Journal of Orthopaedic Research*.

[62] Holst J., Watson S., Lord M. S. (2010). Substrate elasticity provides mechanical signals for the expansion of hemopoietic stem and progenitor cells. *Nature Biotechnology*.

[63] Horiguchi M., Ota M., Rifkin D. B. (2012). Matrix control of transforming growth factor-beta function. *Journal of Biochemistry*.

[64] Robertson I. B., Rifkin D. B. (2016). Regulation of the bioavailability of TGF-beta and TGF-beta-related proteins. *Cold Spring Harbor Perspectives in Biology*.

[65] Taipale J., Miyazono K., Heldin C., Keski-Oja J. (1994). Latent transforming growth factor-beta 1 associates to fibroblast extracellular matrix via latent TGF-beta binding protein. *The Journal of Cell Biology*.

[66] Unsöld C., Hyytiäinen M., Bruckner-Tuderman L., Keski-Oja J. (2001). Latent TGF-beta binding protein LTBP-1 contains three potential extracellular matrix interacting domains. *Journal of Cell Science*.

[67] Wipff P. J., Rifkin D. B., Meister J. J., Hinz B. (2007). Myofibroblast contraction activates latent TGF-*β*1 from the extracellular matrix. *The Journal of Cell Biology*.

[68] Carpenter J., Thomopoulos S., Soslowsky L. (1999). Animal models of tendon and ligament injuries for tissue engineering applications. *Clinical Orthopaedics and Related Research*.

[69] Depres-Tremblay G., Chevrier A., Snow M., Hurtig M. B., Rodeo S., Buschmann M. D. (2016). Rotator cuff repair: a review of surgical techniques, animal models, and new technologies under development. *Journal of Shoulder and Elbow Surgery*.

[70] Derwin K. A., Badylak S. F., Steinmann S. P., Iannotti J. P. (2010). Extracellular matrix scaffold devices for rotator cuff repair. *Journal of Shoulder and Elbow Surgery*.

[71] Liu X., Manzano G., Kim H. T., Feeley B. T. (2011). A rat model of massive rotator cuff tears. *Journal of Orthopaedic Research*.

[72] Lebaschi A., Deng X. H., Zong J. (2016). Animal models for rotator cuff repair. *Annals of the New York Academy of Sciences*.

[73] Sun Y., Kwak J. M., Qi C. (2020). Remnant tendon preservation enhances rotator cuff healing: remnant preserving versus removal in a rabbit model. *Arthroscopy: The Journal of Arthroscopic and Related Surgery*.

[74] Wang C., Hu Q., Song W., Yu W., He Y. (2020). Adipose stem cell-derived exosomes decrease fatty infiltration and enhance rotator cuff healing in a rabbit model of chronic tears. *The American Journal of Sports Medicine*.

[75] Cole B. J., McCarty L. P., Kang R. W., Alford W., Lewis P. B., Hayden J. K. (2007). Arthroscopic rotator cuff repair: prospective functional outcome and repair integrity at minimum 2-year follow-up. *Journal of Shoulder and Elbow Surgery*.

[76] Mook W. R., Greenspoon J. A., Millett P. J. (2016). Arthroscopic double-row transosseous equivalent rotator cuff repair with a knotless self-reinforcing technique. *The Open Orthopaedics Journal*.

[77] Park M. C., Elattrache N. S., Ahmad C. S., Tibone J. E. (2006). “Transosseous-equivalent” rotator cuff repair technique. *Arthroscopy: The Journal of Arthroscopic and Related Surgery*.

[78] Toussaint B., Schnaser E., Bosley J., Lefebvre Y., Gobezie R. (2011). Early structural and functional outcomes for arthroscopic double-row transosseous-equivalent rotator cuff repair. *The American Journal of Sports Medicine*.

[79] Vaishnav S., Millett P. J. (2010). Arthroscopic rotator cuff repair: scientific rationale, surgical technique, and early clinical and functional results of a knotless self-reinforcing double-row rotator cuff repair system. *Journal of Shoulder and Elbow Surgery*.

[80] Gigante A., Bevilacqua C., Ricevuto A., Mattioli-Belmonte M., Greco F. (2007). Membrane-seeded autologous chondrocytes: cell viability and characterization at surgery. *Knee Surgery, Sports Traumatology, Arthroscopy*.

[81] Chen C., Chen Y., Li M. (2020). Functional decellularized fibrocartilaginous matrix graft for rotator cuff enthesis regeneration: a novel technique to avoid in-vitro loading of cells. *Biomaterials*.

[82] Gresham R. C. H., Bahney C. S., Leach J. K. (2021). Growth factor delivery using extracellular matrix-mimicking substrates for musculoskeletal tissue engineering and repair. *Bioactive Materials*.

[83] Porzionato A., Stocco E., Barbon S., Grandi F., Macchi V., De Caro R. (2018). Tissue-engineered grafts from human decellularized extracellular matrices: a systematic review and future perspectives. *International Journal of Molecular Sciences*.

