# Supplementary material for: Engineered Decellularized Tendon Matrix Putty Preserves Native Tendon Bioactivity to Promote Cell Proliferation and Enthesis Repair
Source: J Tissue Eng Regen Med. 2023 Nov 16;2023:4665795. doi: 10.1155/2023/4665795 (PMC11918894; doi:10.1155/2023/4665795)
Supplement: Supplementary Materials — Supplemental Figure 1: (A) male and female donors (n = 5 donors each) on the x-axis displayed by their respective age on the y-axis; (B) no significance was found in total protein between patella and Achilles tendons of male and female donors. Kruskal–Wallis test was performed to measure differences in protein content between male and female tendon types, H(3) = 0.5086, p = 0.917. Supplemental Figure 2: Mann–Whitney tests were performed to find no sex-specific significant differences in (A) TGF-β1, U = 12, p > 0.999, (B) TGF-β2, U = 12, p > 0.999, or (C) TGF-β3, U = 12, p > 0.999, for the male (n = 5 donors) and female (n = 5 donors) donors. (D–F) Additionally, there were no correlations found between age and TGF-β I, II, or III activity. Supplemental Figure 3: Rabbit DTM was tested for (A) total protein content, (B–D) TGFβ activity, and (E) rheological analysis prior to performing in vivo studies. (A–D) Rabbit DTM had significantly more protein content than native rabbit tendon (p = 0.0033). Significantly more TGFβ activity was determined through unpaired two-tailed t-tests (p = 0.0062; p < 0.0001; p < 0.0001, respectively; n = 7 rabbit donors). (E) Rabbit DTM maintained an elastic dominant behavior at all frequencies tested. Supplemental Figure 4: Additional histology images were provided to represent the variance in animals. HBQ images (A–C) were obtained to show collagen (red) and GAGs (blue) at the enthesis. Additional H&E stains (D–F) show differences in the fibrocartilaginous area within enthesis. Scale bars = 100 μm. Supplementary Table 1: Overview of catalog number and vendor where key biological resources were purchased. [file 4665795.f1.docx]

**SUPPLEMENTAL FIGURES**


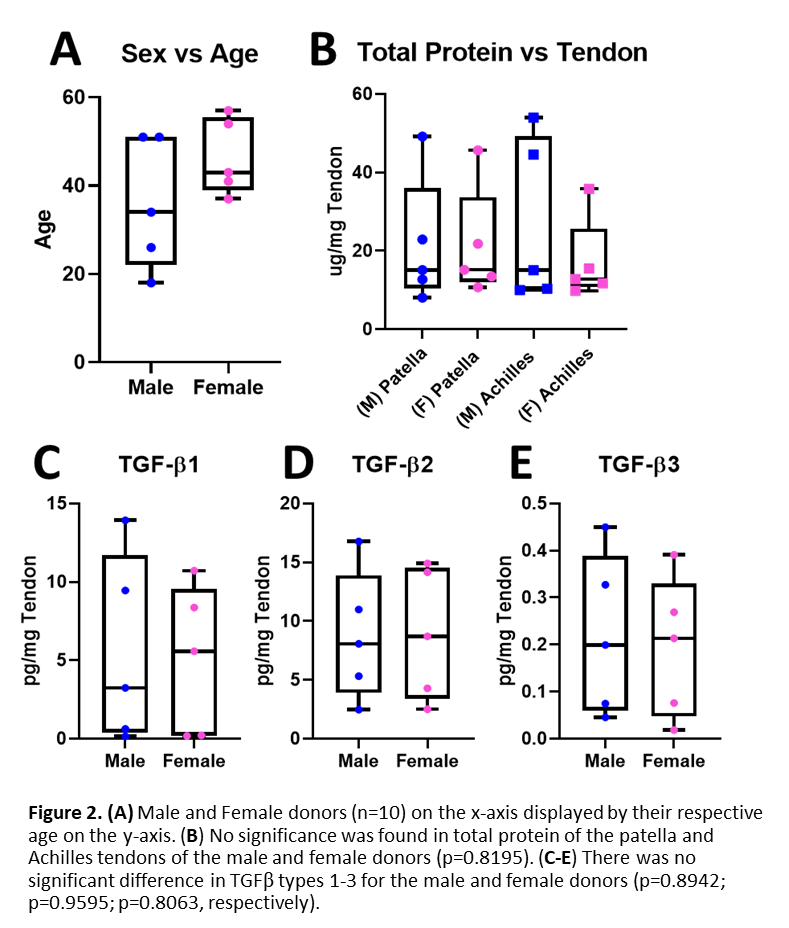


**Supplemental Figure 1.** (**A**) Male and female donors (*n=5 donors each*) on the x-axis displayed by their respective age on the y-axis. (**B**) No significance was found in total protein between patella and Achilles tendons of male and female donors. Kruskal-Wallis test was performed to measure differences in protein content between male and female tendon types, *H(3) = 0.5086, P=0.917*.

**Supplemental Figure 2.** Mann Whitney tests were performed to find no sex specific significant differences in **(A)** TGF-β1, *U=12, p>0.999*, **(B)** TGF-β2, *U=12, p>0.999*, or **(C)** TGF-β3, *U=12, p>0.999*, for the male (*n=5 donors*) and female (*n=5 donors*) donors. **(D-F)** Additionally, there were no correlations found between age and TGF-β I, II or III activity.


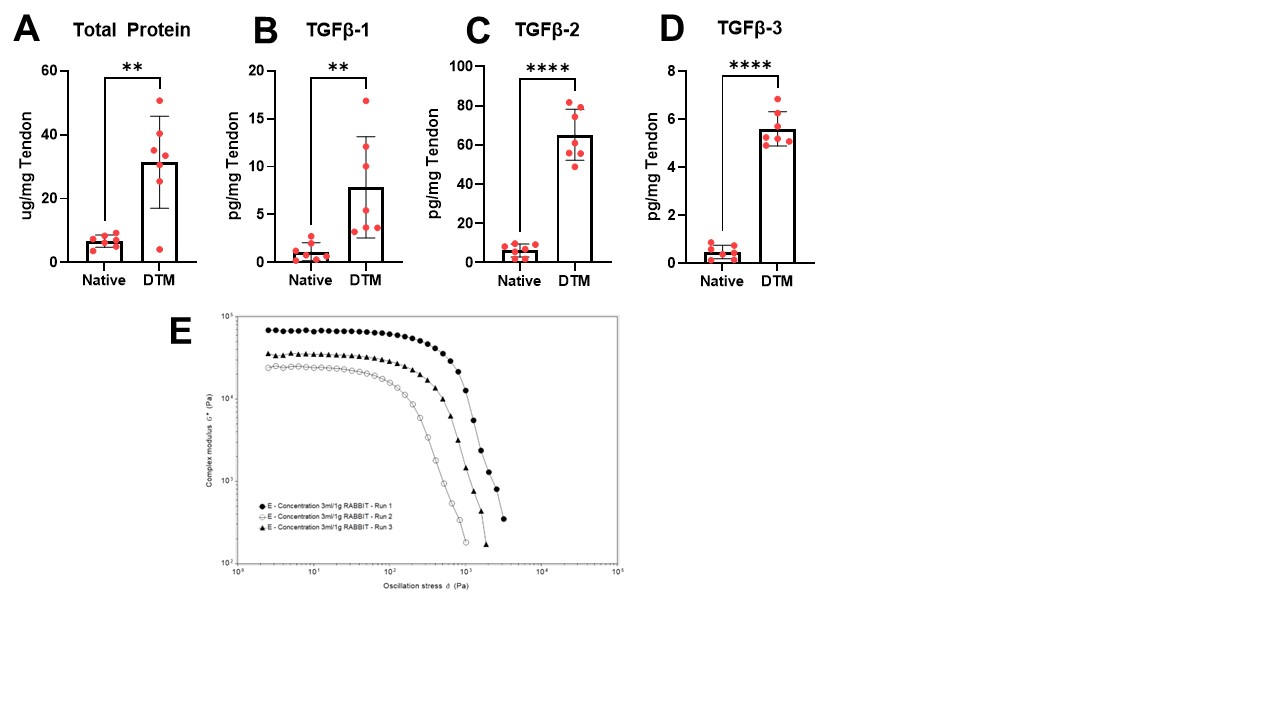
**Supplemental Figure 3.** Rabbit DTM was tested for **A).** total protein content, **B-D).** TGFβ activity and **E).** rheological analysis prior to performing *in vivo* studies. **A-D).** Rabbit DTM had significantly more protein content than native rabbit tendon (*p=0.0033*). Significantly more TGFβ activity was determined through unpaired two-tailed t-tests (*p=0.0062; p<0.0001; p<0.0001* respectively; *n=7* rabbit donors). **E).** Rabbit DTM maintained an elastic dominant behavior at all frequencies tested.


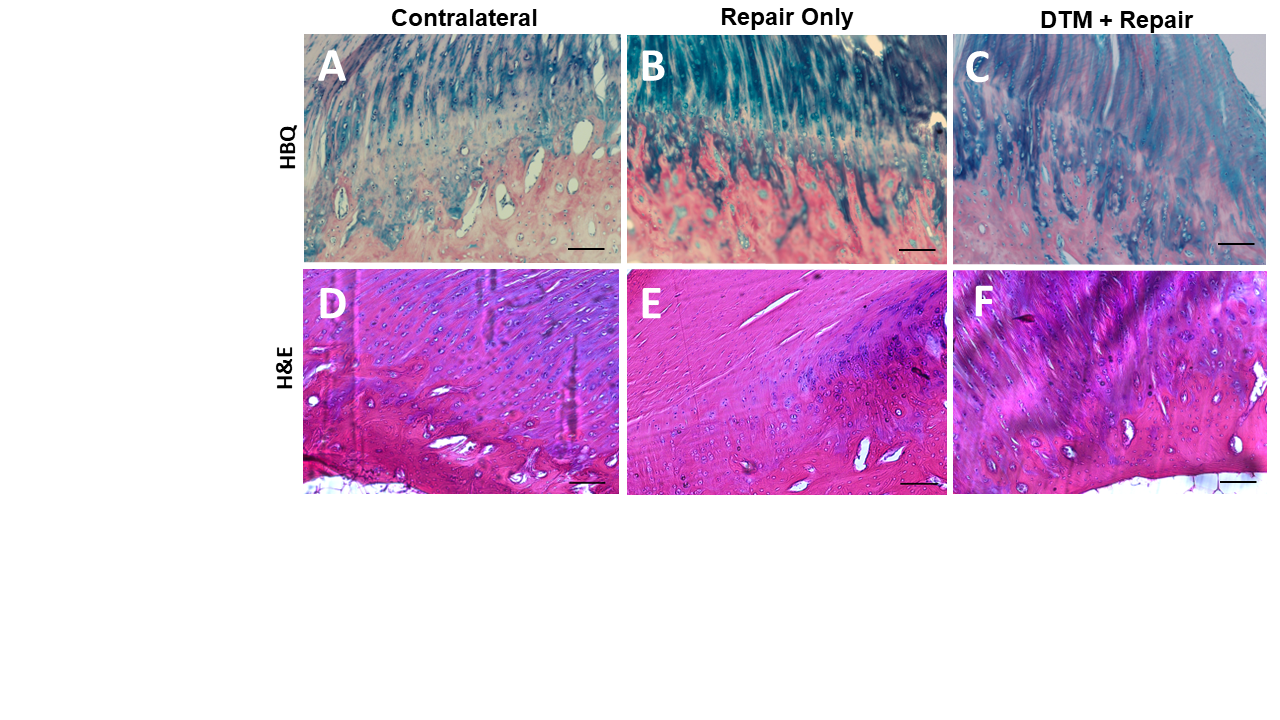
**Supplemental Figure 4.** Additional histology images were provided to represent the variance in animals. HBQ images (**A-C**) were obtained to show collagen (red) and GAGs (blue) at the enthesis. Additional H&E stains (**D-F**) show differences in the fibrocartilaginous area within enthesis. Scale bars = 100 μm.

**Supplementary Table 1.** Overview of catalog number and Vendor where key biological resources were purchased.

| **Resource** | **Vendor** | **Cat #** |
| --- | --- | --- |
| DNEasy Blood & Tissue Kit | Qiagen | 69506 |
| Tecan Infinite 200 Pro Plate Reader | Tecan | 30050303 |
| DNase I | ThermoFisher | EN0521 |
| Collagenase type I | ThermoFisher | 17100017 |
| Collagenase type III | Fisher Scientific | NC9405360 |
| Protein Concentrators 100K MWCO | ThermoFisher | 88503 |
| Collagenase Activity Colorimetric Assay Kit | Abcam | ab196999 |
| T-PER | ThermoFisher | 78510 |
| Protease Inhibitor Cocktail (1X) | Cell Signaling | 1861278 |
| IKA tissue homogenizer | IKA | UX-04720-51 |
| Micro-BCA Kit | ThermoFisher | 23252 |
| TGFꞵ using Milliplex Map TGFβ Magnetic Bead 3 Plex Kit | Millipore Sigma | TGFBMAG-64K-03 |
| Luminex 200™ Instrument | Luminex | LX200-XPON-IVD |
| tenocytes | ZEN-BIO | TEN-F |
| DMEM/F12 | ThermoFisher | 11320033 |
| Fetal Bovine Serum | ThermoFisher | 10437028 |
| Penicillin/streptomycin | Genesee Scientific | 25-512 |
| TGFꞵ1 | PeproTech | 100-21 |
| TGFꞵ2 | PeproTech | 100-35B |
| TGFꞵ3 | PeproTech | 100-36E |
| TGFꞵ inhibitor | SelleckChem | S2704 |
| Collagen-1 coating | Sigma Aldrich | C-9791 |
| Presto Blue Cell Viability Reagent | Thermo Fisher | A13261 |
| Peka Light Engine | Lumencor | 3-NII-FA |
| 4-Nitrophenol (PNP) | Sigma Aldrich | 1048-25G |
| PNP substrate tablets | Sigma Aldrich | N1891 |
| Trizol Lysis Reagent | ThermoFisher | 15596026 |
| qScript cDNA SuperMix | Quanta Bio | 95048 |
| ProFlex PCR System | Applied Biosystems | 4483636 |
| StepOnePlus Real-Time PCR System | Applied Biosystems | 4376592 |
| SYBR Green Master Mix | ThermoFisher | 4368577 |
| Neg-50 | Richard-Allan Scientific | 6502 |
| Fluor shield Mounting Media with DAPI | Abcam | ab104139 |
| Tissue-Tek VIP 6 AI Vacuum Infiltration Processor | Sakura | 6040 |
| VitroView™Picro-Sirius Red Stain Kit | VitroVivo Biotech | VB-3017 |
